# The Effect of Nano-Fertilization With NPK and Spraying With Potassium Silicate on Improving the Physical Characteristics of Date Palm (*Phoenix dactylifera*) Fruits of Khastawi and Khadhrawi Cultivars

**DOI:** 10.1155/sci5/4314722

**Published:** 2025-08-20

**Authors:** Zaidon Abbas Hassan Al-Khafaji, Imad Ali Ubaid Al-Amri

**Affiliations:** ^1^Horticulture Department, Ministry of Agriculture, Baghdad, Iraq; ^2^College of Agriculture, Al-Qasim Green University, Babylon, Iraq

**Keywords:** date palm, foliar spraying, fruit quality, nano NPK, potassium silicate, yield improvement

## Abstract

This study was conducted during the 2024 growing season at the Palm Research Station in Al-Zafaraniya, Baghdad, Iraq. A total of 54 uniform date palm trees (Khastawi and Khadhrawi cultivars), approximately 15 years old and planted at 10-m spacing, were selected for experimental treatments. The objective was to evaluate the effects of three concentrations of nano NPK fertilizer (0, 2, and 4 g·L^−1^), applied in six foliar doses, and three concentrations of potassium silicate (0, 2, and 4 mL·L^−1^), applied in four foliar doses, as well as their interaction, on date yield, and fruit quality. The results revealed significant improvements in all measured traits. Total date yield per tree increased from 29.81 kg in the control to 68.95 kg with the N3S3 treatment. Average bunch weight rose from 7.45 kg to 17.23 kg, and fruit set percentage increased from 66.15% to 79.90%. The Khastawi cultivar produced higher bunch weight and total date yield, whereas Khadhrawi excelled in individual date size and dimensions. The most effective treatment combination was nano NPK at 4 g/L with potassium silicate at 2 mL/L (N3S2), which yielded optimal results across most traits. These findings confirm that the integration of nano-fertilizers and potassium silicate foliar sprays can significantly enhance the productivity and fruit quality of dates under arid environmental conditions.

## 1. Introduction

Because of its substantial nutritional and economic significance, particularly in the Middle East and the Islamic world, the date palm (*Phoenix dactylifera* L.) is one of the most significant plants in the Arecaceae family. It is one of the main economic sectors that contribute to the national economy since it is the main source of extremely nutritious fruits [1].

Due to their early ripening, the Khastawi and Khadhrawi cultivars are two of the most significant date palm types cultivated in central Iraq [2]. With over 19,348,741 date palm trees, Iraq is one of the top date palm-growing nations [2].

But during the previous few decades, date palm productivity has drastically decreased—it has decreased by more than 50%. Date palm productivity is limited by a number of issues, including the poor use of existing agricultural resources, dependence on traditional farming methods, delays in adopting contemporary technology, and limitations in agricultural management practices [3].

Date palm trees' nutritional requirements change according to their growth phases, and they need sufficient amounts of nutrients to maintain their numerous physiological functions. Foliar fertilization is essential in this situation because it provides trees with a balanced supply of nutrients [4].

As a result, scientists have concentrated on creating balanced fertilization plans. This satisfies date palms' fundamental needs, with a particular focus on using nano-fertilizers. Compared to traditional fertilizers, these fertilizers have the advantages of releasing nutrients gradually and sustainably, lowering their loss through soil leaching, and increasing nutrient usage efficiency, which makes them a viable choice for both the environment and the economy [5].

According to studies, using potassium silicate sprays helps plants to withstand harsh weather conditions like drought, salinity, cold, and high heat by lowering water loss [6].

Additionally, studies have shown that applying potassium silicate spray on date palms increases the fruits' physical and chemical properties, bunch weight, and yield amount. In comparison to the control treatment, it also boosts the percentage of fruit set and the amount of chlorophyll and carbohydrates in the leaves [7–9].

In comparison to traditional fertilizers, a number of studies have demonstrated that the use of nano-fertilizers in soil enhances date palm growth and production. According to research findings, nano NPK fertilizer has a good impact on fruit and vegetative features, resulting in increased fresh fruit weight, greater yields, improved fruit quality, and improved nutritional content, including soluble solids and amino acids [10–12].

The purpose of this study is to assess how potassium silicate spraying and nano NPK fertilization, as well as how they interact, might enhance specific fruit physical attributes and boost the yield of Khadhrawi and Khastawi date palm varieties.

## 2. Materials and Methods

### 2.1. Location of the Experiment

The study was conducted during the 2024 growing season at the Zafraniya Date Palm Research Station—Ministry of Agriculture, Baghdad, Iraq, to investigate the role of applying nano NPK fertilizer and spraying with potassium silicate, as well as their interaction, in improving some physical characteristics of the fruits and increasing the yield of date palm trees of the Khadhrawi and Khastawi cultivars. https://maps.app.goo.gl/kbtAehLbmMb4mBgk8.

### 2.2. The Preparation of the Orchard and Agricultural Methods

Al-Zafraniya Date Palm Station covers an area of 62 dunums and contains approximately 5000 date palm trees, which were planted in 2010 with a spacing of 10 × 10 m between trees. The orchard is irrigated using a modern drip irrigation system to ensure efficient water use and minimize losses. An integrated fertilization system is followed at the station, combining organic fertilization using sheep manure with chemical fertilization using a balanced NPK fertilizer, aiming to enhance tree growth and improve both the quality and quantity of production.

Fifty-four date palm trees of the Khastawi and Khadhrawi varieties, which were at least 15 years old and as uniform in height and age as feasible, were chosen. Drip irrigation was used to water the trees, which were planted in rows 10 m apart and divided among three replicates, each of which included 18 experimental treatments. Every tree served as a unit of experimentation. Pruning; clearing away dry fronds, offshoots, and suckers; controlling pests and insects; watering; maintaining the drip irrigation system; and maintaining the crown—which included pollination using the green Al-Ghannami pollinator when the female inflorescence opened, thinning, bunch thinning, and tying—were all examples of agricultural operations.

### 2.3. Experimental Treatments

The study included three factors:

• Factor 1: Two date palm cultivars, Khastawi and Khadhrawi.
• Factor 2: Spraying potassium silicate at three different concentrations (0, 2, and 4 mL·L^−1^) and adding 1 mL·L^−1^ of the surfactant Tween 20 to the solution will lower the water's surface tension and improve the solution's adherence to the leaves and fruits, which will improve absorption. A 400-L mechanical pump was used to spray the trees early in the morning, applying 15 L to each tree until they were completely saturated. The experiment's designated addition intervals were followed while repeating the spraying procedure. The addition was made in four doses: 4 weeks after pollination, 8 weeks after pollination, 12 weeks after pollination, and before the female inflorescences opened on March 10.
• Factor 3: Three concentrations of nano NPK fertilizer (0, 2, 4 g·L^−1^) were applied in six doses: 5 weeks after pollination, 6 weeks after pollination, 9 weeks after pollination, 12 weeks after pollination, and 16 weeks after pollination. The first dose was applied prior to the opening of the female inflorescences on March 10.

### 2.4. Experimental Design

The study was conducted as a factorial experiment (3 × 3 × 2) with a completely randomized block design (RCBD) and three replicates. Each replicate consisted of 18 trees, with one tree per experimental unit, resulting in a total of 54 trees. The results were analyzed using the ANOVA table in the Genstat 2012 program. Statistical differences between treatments were tested using the least significant difference (LSD) test at a 0.05 probability level [13]. The traits that were studied include:

### 2.5. Traits Under Study

#### 2.5.1. Physical Traits of the Fruits

The physical traits of the date fruits of the Khastawi and Khadhrawi cultivars were measured as follows:

1. Fruit set percentage (%): This percentage was calculated one month after pollination by randomly selecting 10 spikes from each bunch. The number of set fruits and the number of empty scars were counted according to the following equation:
  (1) $$\begin{array}{c} \text{fruit set percentage}\left(\%\right)=\frac{\text{number of set fruits}}{\text{number of set fruits}+\text{number of empty scars}}\times 100. \end{array}$$
2. Fruit length (cm): Fruit length was measured using a measuring tape by randomly selecting 20 fruits from each replicate and treatment. The measurements were conducted during the advanced Khalal stage, around the first week of August, when the fruits had reached physiological maturity and full size.
3. Fruit diameter (cm): The diameter of the fruits was measured at the widest point using a Vernier caliper. Twenty fruits were randomly selected from each replicate and treatment, and the average diameter was calculated. Measurements were carried out during the advanced Khalal stage, around the first week of August, when the fruits had reached their full size and physiological maturity.
4. Fruit volume (cm^3^ per fruit): Fruit volume was determined using the water displacement method. Twenty fruits were randomly selected from each replicate and treatment and immersed in a graduated cylinder containing a known initial volume of water. After submerging the fruits, the final water level was recorded, and the volume of displaced water was calculated as the difference between the initial and final readings. The average volume per fruit was obtained by dividing the total displaced volume by the number of fruits. This measurement was conducted during the advanced Khalal stage, around the first week of August, when the fruits had reached their final size and physiological maturity.
  (2) $$\begin{array}{c} \text{fruit volume}\left({\text{cm}}^{3}\right)=\frac{\text{total fruit volume}}{\text{number of fruits}}. \end{array}$$
5. Average fruit weight (g): The fresh weight of the selected fruits was measured using a precision digital scale. A total of 20 fruits were randomly selected from each replicate and treatment, and their combined weight was recorded. The average fresh fruit weight was then calculated by dividing the total weight by the number of fruits. This measurement was carried out during the advanced Khalal stage, approximately in the first week of August, when the fruits had reached their full size and physiological maturity.
  (3) $$\begin{array}{c} \text{average fruit weight}\left(g\right)=\frac{\text{total weight of fruits}}{\text{total number of fruits}}. \end{array}$$
6. Average seed weight (g): Seed kernels were carefully removed from 20 randomly selected fruits per replicate and treatment. The combined mass of all extracted seeds was determined using a precision balance, and the mean seed weight was calculated by dividing the total seed mass by the number of seeds. All measurements were performed during the advanced Khalal stage, approximately in the first week of August, when the fruits had completed physiological maturation.
7. Average flesh weight (g): The average pericarp (flesh) weight was determined by subtracting the mean seed weight from the mean total fruit weight. Measurements were conducted during the advanced Khalal stage, approximately in the first week of August, when fruits had attained full size and physiological maturity.
8. Average bunch weight (kg): The average bunch weight for each treatment was calculated by measuring the weight of the marked bunches and dividing by the number of bunches.
9. Total yield (kg per tree): Total fruit yield per tree and per replicate was quantified at harvest by weighing all bunches with a field scale. The aggregate yield weight was then averaged across trees to obtain the mean total yield. All measurements were conducted at the commercial maturity stage (Tamar), during the harvest period in late August to early September, when fruit moisture content and sugar accumulation had stabilized.
10. Fruit ripening percentage (%): Ten spikes were randomly selected from each bunch during the ripe stage. The number of ripe fruits and the total number of fruits in the selected spikes were counted. The ripening percentage was calculated using the following equation:
  (4) $$\begin{array}{c} \text{ripening percentage}\left(\%\right)=\frac{\text{number of ripe fruits}}{\text{total number of fruits}}\times 100. \end{array}$$

## 3. Results

### 3.1. Fruit Set Percentage (%)

The results demonstrated a significant impact of cultivar type on enhancing fruit set percentage, with the Khastawi cultivar (V1) achieving an average fruit set of 75.64%, compared to 70.58% recorded for the Khadhrawi cultivar (V2) (Table 1).

With an average fruit set of 75.64%, the S3 (4 mL·L^−1^) treatment (potassium silicate spraying) outperformed the S1(0) treatment (control), which had a 71.22% (Table 1).

With a fruit set percentage of 74.55 after the addition of nano NPK, the N3 (4 g·L^−1^) treatment outperformed the N2 (2 g·L^−1^) treatment, which had the lowest percentage at 71.86.

According to the results of the factor interaction, the N3S3 treatment had the greatest fruit set percentage (76.91), whereas the N2S1 treatment had the lowest percentage (69.72) (Table 1).

The V1S3 treatment had the highest fruit set percentage (77.82) in relation to the interaction between cultivar type and potassium silicate spraying, whereas the V2S1 treatment had the lowest percentage (68.96) (Table 1). The V1N3 treatment had the greatest fruit set percentage (77.76) in the interaction between nano NPK and cultivar type, whereas the V2N2 treatment had the lowest (68.74) (Table 1). The V1N3S3 treatment had the highest fruit set percentage (79.90), and the V2N2S1 treatment had the lowest (66.15), according to the triple interaction of cultivar type, potassium silicate spraying, and nano NPK addition (Table 1).

### 3.2. Fruit Length (cm)

The Khadhrawi cultivar (V2) outperformed the Khastawi cultivar (V1), recording an average of 3.65 cm compared to 3.33 cm, indicating a substantial impact of cultivar variations on fruit length (Table 2).

Furthermore, the findings showed that potassium silicate spraying had a substantial impact; the S3 (4 mL·L^−1^) treatment had the greatest value, measuring 3.70 cm, while the control treatment S1(0) had the lowest, measuring 3.26 cm (Table 2).

When nano NPK was added, the N2 (2 g·L^−1^) treatment had the highest average value (3.59 cm), followed by the N3 (4 g·L^−1^) treatment (3.58 cm), but there was no discernible difference between the two (Table 2). When it came to the factor interaction, the N3S3 treatment had the longest fruit, measuring 3.82 cm, whereas the N1S1 treatment only had 3.07 cm (Table 2). Regarding the relationship between cultivar type and potassium silicate spraying, the V2S3 treatment had the greatest value (3.90 cm), while the V1S1 treatment had the lowest value (3.08 cm) (Table 2).

When nano NPK and cultivar type interacted, the V2N2 treatment had the highest average (3.88), the V2N1 treatment the lowest (3.26), and the V1N2 treatment the third (3.29) (Table 2). Last but not least, the V2N3S3 treatment had the greatest value of 4.18 cm with the triple interaction of cultivar type, potassium silicate spraying, and nano NPK addition, whereas the V1N2S1 treatment had the lowest value of 3.01 cm (Table 2).

### 3.3. Fruit Diameter (cm)

The findings demonstrated that variety variations significantly affected the average fruit diameter, with the Khadhrawi variety (V2) recording an average diameter of 1.76 cm and the Khastawi variety (V1) recording 1.68 cm (Table 3).

When it came to potassium silicate spraying, treatment S2 (2 mL·L^−1^) had the highest average, measuring 1.81 cm, whereas treatment S1(0), the control, recorded 1.60 cm (Table 3).

The greatest average of 1.77 cm for nano NPK addition was obtained with treatment N3 (4 g·L^−1^), which was followed by treatment N2 (2 g·L^−1^) with an average of 1.69 cm (Table 3). Treatment N1 (0 g/L) had an average of 1.70 cm and showed no discernible changes. In contrast to the S1N2 treatment, which recorded 1.53 cm, the S2N3 treatment recorded the highest average of 1.86 cm, according to the interaction findings between the components (Table 3). Treatment V2S2 had the greatest average of 1.83 cm in the interaction between variety type and potassium silicate spraying, whereas treatment V1S1 had the lowest average of 1.54 cm (Table 3). Treatment V2N3 recorded the greatest average of 1.85 cm for the interaction between variety type and nano NPK addition, whereas treatment V1N2 recorded 1.67 cm (Table 3). The average for treatment V1N1 was 1.68 cm. The V2S2N3 treatment had a largest average of 1.97 cm, but the V1S1N2 treatment had the lowest average of 1.47 cm, due to the triple interaction of variety type, potassium silicate spraying, and nano NPK addition (Table 3).

### 3.4. Fruit Volume (cm^3^ per Fruit)

With an average of 7.45 cm^3^ compared to 6.99 cm^3^, the Khadhrawi cultivar (V2) outperformed the Khastawi cultivar (V1), demonstrating a substantial impact of cultivar differences on average fruit volume (Table 4).

When it came to potassium silicate spraying, the S2 treatment (2 mL·L^−1^) produced the largest average fruit volume (7.98 cm^3^), whereas the control treatment S1(0) produced 6.06 cm^3^ (Table 4).

Regarding the addition of nano NPK, the control treatment N1(0) had the smallest average fruit volume at 6.17 cm^3^, whereas the N3 treatment (4 g·L^−1^) had the largest at 8.07 cm^3^. The S2N3 treatment produced the largest average fruit volume (9.15 cm^3^), whereas the S1N1 treatment produced the smallest at 5.75 cm^3^, according to the results of the factor interaction (Table 4).

When cultivar and potassium silicate spraying were combined, the V2S2 treatment (Khadhrawi + 2 mL/L potassium silicate) produced the largest average fruit volume (8.54 cm^3^), whereas the V1S1 treatment (Khastawi + control) produced the smallest (5.96 cm^3^) (Table 4). The V2N3 treatment (Khadhrawi + 4 g/L nano NPK) had the greatest average fruit volume of 8.54 cm^3^ for the interaction between cultivar and nano NPK addition, whereas the V2N1 treatment (Khadhrawi + 0 g/L nano NPK) had the lowest at 6.00 cm^3^ (Table 4). The V2S2N3 treatment (Khadhrawi + 2 mL/L potassium silicate + 4 g/L nano NPK) had the highest average fruit volume at 10.23 cm^3^, while the V2S1N1 treatment (Khadhrawi + control + 0 g/L nano NPK) had the lowest at 5.57 cm^3^ (Table 4). This was the result of the triple interaction between cultivar, potassium silicate spraying, and nano NPK addition (Table 4).

### 3.5. Average Fruit Weight (g)

The findings revealed notable variations in fruit weight among the cultivars, with the Khadhrawi cultivar (V2) averaging 12.23 g and the Khastawi cultivar (V1) recording 11.90 g (Table 5).

When it came to potassium silicate spraying, the S2 (2 mL·L^−1^) treatment had the greatest average fruit weight (12.95 g), whereas the S1(0) control treatment had the lowest average fruit weight (10.78 g) (Table 5). The N3 (4 g·L^−1^) treatment produced the greatest average fruit weight of 12.92 g after adding nano NPK, whereas the N1(0) control treatment produced the lowest weight of 10.98 g (Table 5). The S2N3 treatment had the greatest average fruit weight, 14.28 g, according to the two-factor interaction results, whereas the S1N1 treatment had the lowest, 10.57 g (Table 5). The V1S1 treatment recorded the lowest average fruit weight of 10.68 g, while the V2S2 treatment attained the greatest average fruit weight of 13.42 g in the interaction between cultivar type and potassium silicate spraying (Table 5). In the interaction between cultivar type and nano NPK addition, the V2N3 treatment produced the highest average fruit weight (13.43 g) in contrast to the V2N1 treatment (10.71 g) (Table 5). The V2S2N3 treatment had the highest average fruit weight (15.35 g) in the three-way interaction between cultivar type, potassium silicate spraying, and nano NPK addition, whereas the V2S1N1 treatment had the lowest weight (10.39 g) (Table 5).

### 3.6. Average Seed Weight (g)

The findings demonstrated that the conditions under study had a considerable impact on seed weight at the Tamr stage (Table 6).

The Khadhrawi cultivar (V2) reported a seed weight of 0.71 g, whereas the Khastawi cultivar (V1) had a greater seed weight of 0.72 g (Table 6). In terms of potassium silicate spraying, the treatments S3 (4 mL·L^−1^) and S2 (2 mL·L^−1^) had the greatest seed weights, weighing 0.73 g as opposed to 0.69 g for the control S1(0) (Table 6). Treatment N3 (4 g·L^−1^) recorded the maximum seed weight of 0.73 g for nano NPK addition, whereas treatment N2 (2 g·L^−1^) recorded the lowest weight of 0.69 g (Table 6). The maximum seed weight, 0.79 g, was recorded by treatment S3N3, whereas treatment S1N2 recorded 0.63 g, according to the two-factor interaction results (Table 6). Treatments V2S3 and V1S2 had the greatest seed weights (0.73) in the interaction between cultivar type and potassium silicate spraying, whereas treatment V2S1 had the lowest weight (0.68%) (Table 6). With respect to the relationship between cultivar type and nano NPK addition, treatment V2N3 had the greatest seed weight of 0.73 g, matching V2N1 and V1N3 (Table 6). Treatment V2S3N3 had the maximum seed weight of 0.79 g for the three-factor interaction, whereas V2S1N2 had the lowest weight of 0.57 g (Table 6).

### 3.7. Average Flesh Weight (g)

The Khadhrawi (V2) variety weighed 11.51 g more than the Khastawi (V1) variety, which weighed 11.31 g (Table 7).

The results demonstrated a considerable impact of variety variations on the fruit flesh weight. Treatment S2 (2 mL·L^−1^) weighed the most (12.22 g) in relation to potassium silicate spraying, whereas treatment S1(0), the control, weighed the least (10.12 g) (Table 6).

Treatment N3 (4 g·L^−1^) had the maximum weight (12.18 g) with the addition of nano NPK, whereas treatment N1(0) (control) had the lowest weight (10.27 g) (Table 7). According to the findings of the fertilizer treatment interaction, S2N3 combined with S1N1 produced the maximum weight (13.54 g), whereas S1N1 produced the lowest weight (9.83 g) (Table 7). The treatment V2S2 had the maximum weight (12.69 g) in the interaction between potassium silicate spraying and variety, whereas V1S1 had the lowest weight (10.04 g) (Table 7). In terms of the relationship between variety and nano NPK addition, treatment V2N3 had the maximum weight (12.70 g) in contrast to V2N1 (9.99 g) (Table 7). The treatment V2S2N3 had the maximum fruit flesh weight of 14.64 g in the triple interaction between the components, whereas V2S1N1 had the lowest weight of 9.63 g (Table 7).

### 3.8. Average Bunch Weight (kg)

With a weight of 13.65 kg, the Khastawi cultivar (V1) outperformed the Khadhrawi cultivar (V2), which recorded 12.37 kg, the results demonstrated a considerable impact of cultivar variations on bunch weight (Table 8).

The control treatment, S1(0), recorded the lowest weight of 10.55 kg, whereas the S3 (4 mL·L^−1^) treatment recorded the maximum weight of 14.26 kg in relation to potassium silicate spraying (Table 8). The control treatment N1(0) recorded the lowest weight of 10.29 kg, while the N3(4 g·L^−1^) treatment displayed the maximum weight of 14.97 kg for the nano NPK addition (Table 8). The S2N3 treatment had the highest weight (16.55 kg) compared to the S1N1 treatment (7.94 kg), according to the results of the interaction between fertilization factors (Table 8). The V1S3 treatment had the maximum weight (15.46 kg) in the interaction between cultivar and potassium silicate spraying, whereas the V2S1 treatment had the lowest weight (10.16 kg) (Table 8). In terms of the relationship between cultivar and nano NPK addition, the V1N3 treatment performed better than the V2N1 treatment, weighing 15.46 kg as opposed to 9.55 kg (Table 8). The V1S3N3 treatment recorded the greatest weight (17.23 kg) in the triple interaction between the variables, whereas the V2S1N1 treatment recorded the lowest weight (7.45 kg) (Table 8).

### 3.9. Total Yield (kg per Tree)

The Khastawi cultivar (V1) produced 54.57 kg, whereas the Khadhrawi cultivar (V2) produced 49.48 kg, indicating a considerable cultivar influence on the overall yield (Table 9).

Regarding the impact of potassium silicate spraying, the control treatment S1(0) gave the lowest amount at 42.13 kg, while the treatment S3 (4 mL·L^−1^) produced the largest amount at 57.06 kg (Table 9). When nano NPK was added, the treatment N3 (4 g·L^−1^) yielded the most (59.88 kg), whereas the control treatment N1(0) yielded the least (41.18 kg) (Table 9). The treatment S2N3 produced the maximum yield (66.21 kg) in the dual interaction between the fertilization factors, while the treatment S1N1 produced the lowest yield (31.76 kg) (Table 9). The treatment V1S3 produced the largest yield of 61.83 kg in the interaction between cultivar type and potassium silicate spraying, whereas V2S1 produced 40.62 kg (Table 9). In terms of the relationship between cultivar type and nano NPK addition, treatment V1N3 produced the maximum yield at 61.85 kg, whereas V2N1 produced 38.20 kg (Table 9). The treatment V1S3N3 produced the maximum yield (68.95 kg) in the triple interaction between the variables, whereas V2S1N1 produced the lowest yield (29.81 kg) (Table 9).

### 3.10. Fruit Ripening Percentage (%)

According to the findings, the percentage of mature plants increased significantly across cultivars (Table 10).

With a maturity percentage of 84.89%, the cultivar Khastawi (V1) had the highest percentage, followed by Khadhrawi (V2) with 83.67% (Table 10). The treatment S3 (4 mL·L^−1^) yielded the greatest maturity percentage of 90.22% in relation to the impact of potassium silicate spraying, while the control treatment S1(0) recorded 77.33% (Table 10). Among the control treatments for nano NPK addition, N1(0) had the greatest maturity percentage (89.50%), whereas N3 (4 g·L^−1^) had the lowest (81.50%) (Table 10). In the dual interaction between the fertilization factors, S3N1 produced the highest maturity percentage (95.83%), whereas S1N3 produced the lowest (71.83%) (Table 10). The treatment V1S3 produced the highest maturity percentage (91.22%) in the interaction between cultivar type and potassium silicate spraying, whereas V2S1 recorded 76.45% (Table 10). The treatment V2N1 had the greatest maturity percentage (90.00%) for the interaction between nano NPK addition and cultivar type, whereas V2N3 recorded 80.11% (Table 10). In comparison to V2S1N3, which recorded the lowest percentage of 70.33%, the treatment V2S3N1 delivered the maximum maturity percentage of 96.33% in the triple interaction between variables (Table 10).

## 4. Discussion

### 4.1. Physiological Effects of Potassium Silicate on Fruit Physical Traits

The application of potassium silicate significantly improved key fruit traits—such as diameter, size, total weight, pulp weight, and seed weight—especially at the S2 concentration (2 mL/L). These enhancements are attributed to the dual physiological actions of potassium (K) and silicon (Si), both of which play fundamental roles in plant growth and fruit development. Silicon improves root growth and enhances water and nutrient uptake, increasing the palm's tolerance to drought and salinity stresses [9, 14, 15]. It also regulates stomatal behavior, thereby reducing transpiration and improving water-use efficiency [14, 16]. Additionally, silicon enhances photosynthetic capacity by promoting CO_2_ absorption and increasing chlorophyll content [17–19]. Its role in cell wall reinforcement supports better cell elongation and differentiation in meristematic regions, contributing to fruit enlargement [14, 20]. These structural effects also improve fruit firmness and resistance to mechanical damage during postharvest handling [14, 16, 17].

### 4.2. Role of Nano NPK in Enhancing Yield and Fruit Physical Characteristics

Foliar application of nano NPK at 4 g/L (N3) significantly improved fruit weight, size, pulp weight, and bunch yield. These outcomes stem from the high reactivity and bioavailability of nano-formulated nutrients. The nanoscale size facilitates enhanced root absorption and translocation of nitrogen (N), phosphorus (P), and potassium (K) [21–23]. Nano nitrogen promotes chlorophyll biosynthesis, increasing photosynthetic efficiency and energy production for fruit development [24, 25]. Nano phosphorus enhances nucleic acid and ATP synthesis, supporting active cell division and elongation [24–26]. Nano potassium facilitates sugar transport and carbohydrate translocation into fruits, improving their weight and size [8, 20, 26]. The controlled-release nature of nano NPK also reduces nutrient losses via leaching or volatilization, ensuring sustained availability throughout fruit development [23, 27]. Furthermore, nano NPK stimulates metabolic enzymes and meristematic activity, enhancing fruit tissue differentiation and physiological ripening [11, 24, 25].

### 4.3. Potassium Silicate and Nano NPK Interaction

The combination of spraying potassium silicate and adding nano NPK produced favorable outcomes, as the combined treatments (like S2N3 and V2S2N3) greatly enhanced all fruit physical characteristics, including overall fruit maturity. This implies a beneficial relationship between potassium silicate and nano NPK in terms of fostering development and raising output.

### 4.4. The Findings Demonstrated That the Physical Characteristics of the Fruit Were Affected Differently by Potassium Silicate and Nano NPK Concentrations

Moderate amounts (S2 and N3), for instance, were the most efficient in enhancing physical characteristics and production; extremely high or very low quantities were less effective. In order to get the finest outcomes, it is crucial to figure out the ideal concentration.

### 4.5. Cultivar Response Differences

While both Khastawi (V1) and Khadhrawi (V2) cultivars showed improvements across the evaluated traits in response to nano NPK and potassium silicate treatments, the magnitude of these improvements varied between the cultivars and among the different traits. For example, in terms of total yield, Khastawi exhibited a 56.3% increase (from 33.71 kg in V1S1N1 to 68.95 kg in V1S3N3), whereas Khadhrawi increased by only 56.3% (from 29.81 kg to 46.67 kg) over the same treatment range—indicating similar responsiveness in relative terms. However, the increase in average fruit weight was 42.7% in Khadhrawi (from 10.39 g to 15.35 g) versus 22.8% in Khastawi (from 10.76 g to 13.26 g), showing greater relative sensitivity of Khadhrawi to fertilization in that trait. Likewise, the increase in fruit diameter in Khastawi under NPK and silicate treatments was about 13.7% (from 1.47 cm to 1.76 cm), while Khadhrawi improved by 23.1% (from 1.60 cm to 1.97 cm). This suggests that Khadhrawi, although recording lower absolute values in some traits, responded more markedly in proportional terms, especially in physical dimensions like fruit length and diameter. The variability in responsiveness suggests genotype-specific nutrient-use efficiencies and physiological thresholds. Therefore, while Khastawi generally outperformed Khadhrawi in absolute yield and fruit set, Khadhrawi demonstrated a more pronounced relative improvement in several traits, implying that the two cultivars differ not only in baseline performance but also in their plasticity and responsiveness to exogenous inputs. This implies that different tree kinds may respond differently to the treatments, highlighting the significance of choosing the right varieties that stand to gain the most from the therapies under study.

### 4.6. Preliminary Economic Profitability Analysis

A simplified economic evaluation was conducted to assess the profitability of the different fertilization treatments. The treatment N3S2 (nano NPK at 4 g/L + potassium silicate at 2 mL/L) exhibited the highest net profit compared to all other treatments, achieving approximately 45% greater net return than the control (N1S1). This increase is primarily attributed to the significant improvement in total yield (reaching up to 68.95 kg/tree) and enhanced fruit quality parameters. Although a comprehensive cost–benefit analysis was beyond the scope of this study, preliminary findings underscore the economic advantage of integrated nano fertilization and silicon foliar application. A summary of cost indicators, estimated revenues, and net income per treatment is presented in Table 11. Future studies should incorporate detailed economic assessments under real farm conditions to better quantify cost-effectiveness across different treatment levels.

Future work should include water use efficiency (WUE) as a key performance indicator to assess whether nano NPK and potassium silicate treatments contribute to enhanced water uptake or retention in date palm cultivation systems.

## 5. Conclusion

The development of date palm plants and the enhancement of fruit production attributes are positively impacted by both applying potassium silicate spray and nano NPK.

Fruit quality and output may be greatly increased by figuring out the ideal concentrations for each. These results are in line with research in References [6, 8, 9, 28, 29] in which it is discovered that giving date palm trees a potassium silicate spray increased the percentage of fruit set, which in turn increased yield and improved the fruit's physical and quality characteristics when compared to the control treatment. These results are also consistent with research in References [10–12], which showed that supplementing date palms with nano NPK fertilizer improved the fruit's physical characteristics and growth rate, leading to higher yields and higher-quality dates.

Although this study was conducted over a single season, the uniform agricultural conditions and precise experimental design yielded statistically significant and consistent results. The observed treatment effects followed clear biological trends, suggesting true physiological responses rather than random environmental variation. Moreover, the applied materials (nano NPK and potassium silicate) are known for their stable physiological impact, which lends further credibility to the findings. The consistency and clarity of responses across traits support the validity of the conclusions despite the single-season scope. Nonetheless, we strongly recommend conducting multiseasonal studies to confirm and strengthen these findings under varying environmental conditions.

## Figures and Tables

**Table 1:** Effect of nano NPK addition and potassium silicate spraying on fruit set percentage.

V cultivars	NPK nano (g·L^−1^)	Potassium silicate (mL·L^−1^)			V × N	Avg V
		(0)S1	(2)S2	(4)S3		
Khastawi V1	(0)N1	72.82	72.83	76.65	74.18	75.64
	(2)N2	73.28	74.99	76.65	74.97	
	(4)N3	74.34	79.05	79.90	77.76	

V2 Khadhrawi	(0)N1	72.12	68.66	74.23	71.67	70.58
	(2)N2	66.15	69.31	70.76	68.74	
	(4)N3	68.61	71.47	73.91	71.33	

L.S.D 0.05		0.10462			0.06040	0.03487

V × S	V1	73.48	75.62	77.82	L.S.D 0.05	
	V2	68.96	69.81	72.96	0.06040	

N × S	(0)N1	72.47	70.74	75.56	Avg N	72.93
	(2)N2	69.72	72.15	73.70		71.86
	(4)N3	71.48	75.26	76.91		74.55

0.07398	Avg S	71.22	72.72	75.39	0.04271	L.S.D 0.05

**Table 2:** Effect of nano NPK addition and potassium silicate spraying on fruit length (cm).

V cultivars	NPK nano (g·L^−1^)	Potassium silicate (mL·L^−1^)			V × N	Avg V
		(0)S1	(2)S2	(4)S3		
Khastawi V1	(0)N1	3.08	3.33	3.60	3.34	3.33
	(2)N2	3.01	3.41	3.45	3.29	
	(4)N3	3.17	3.45	3.47	3.36	

V2 Khadhrawi	(0)N1	3.05	3.23	3.49	3.26	3.65
	(2)N2	3.59	4.02	4.04	3.88	
	(4)N3	3.51	3.69	4.18	3.79	

L.S.D 0.05		0.01420			0.00820	0.00473

V × S	V1	3.08	3.39	3.50	L.S.D 0.05	
	V2	3.38	3.64	3.90	0.00820	

N × S	(0)N1	3.07	3.28	3.55	Avg N	3.30
	(2)N2	3.30	3.71	3.75		3.59
	(4)N3	3.43	3.49	3.82		3.58

0.01004	Avg S	3.26	3.49	3.70	0.00580	L.S.D 0.05

**Table 3:** Effect of nano NPK addition and potassium silicate spraying on fruit diameter (cm) for the 2024 season.

V cultivars	NPK nano (g·L^−1^)	Potassium silicate (mL·L^−1^)			V × N	Avg V
		(0)S1	(2)S2	(4)S3		
Khastawi V1	(0)N1	1.56	1.78	1.70	1.68	1.68
	(2)N2	1.47	1.81	1.73	1.67	
	(4)N3	1.58	1.75	1.76	1.70	

V2 Khadhrawi	(0)N1	1.64	1.70	1.79	1.71	1.76
	(2)N2	1.60	1.82	1.74	1.72	
	(4)N3	1.73	1.97	1.83	1.85	

L.S.D 0.05		0.011264			0.006503	0.00376

V × S	V1	1.54	1.78	1.73	L.S.D 0.05	
	V2	1.66	1.83	1.79	0.06503	

N × S	(0)N1	1.60	1.74	1.75	Avg N	1.70
	(2)N2	1.53	1.81	1.73		1.69
	(4)N3	1.66	1.86	1.80		1.77

0.007965	Avg S	1.60	1.81	1.76	0.004598	L.S.D 0.05

**Table 4:** Effect of nano NPK addition and potassium silicate spraying on average fruit volume (cm^3^) for the 2024 season.

V cultivars	NPK nano (g·L^−1^)	Potassium silicate (mL·L^−1^)			V × N	Avg V
		(0)S1	(2)S2	(4)S3		
Khastawi V1	(0)N1	5.93	6.77	6.33	6.34	6.99
	(2)N2	5.80	7.40	7.87	7.02	
	(4)N3	6.13	8.07	8.57	7.59	

V2 Khadhrawi	(0)N1	5.57	6.00	6.43	6.00	7.45
	(2)N2	6.23	9.40	7.77	7.80	
	(4)N3	6.67	10.23	8.73	8.54	

L.S.D 0.05		0.01989			0.01148	0.0066

V × S	V1	5.96	7.41	7.59	L.S.D 0.05	
	V2	6.16	8.54	7.64	0.01148	

N × S	(0)N1	5.75	6.38	6.38	Avg N	6.17
	(2)N2	6.02	8.40	7.82		7.41
	(4)N3	6.40	9.15	8.65		8.07

0.01406	Avg S	6.06	7.98	7.62	0.00812	L.S.D 0.05

**Table 5:** Effect of nano NPK addition and potassium silicate spraying on average fruit weight (g) for the 2024 season.

V cultivars	NPK nano (g·L^−1^)	Potassium silicate (mL·L^−1^)			V × N	Avg V
		(0)S1	(2)S2	(4)S3		
Khastawi V1	(0)N1	10.76	11.66	11.34	11.25	11.90
	(2)N2	10.48	12.59	13.03	12.03	
	(4)N3	10.80	13.21	13.26	12.42	

V2 Khadhrawi	(0)N1	10.39	10.71	11.04	10.71	12.23
	(2)N2	10.86	14.21	12.61	12.56	
	(4)N3	11.39	15.35	13.54	13.43	

L.S.D 0.05		0.02043			0.01179	0.0068

V × S	V1	10.68	12.48	12.54	L.S.D 0.05	
	V2	10.88	13.42	12.40	0.01179	

N × S	(0)N1	10.57	11.18	11.19	Avg N	10.98
	(2)N2	10.67	13.40	12.82		12.30
	(4)N3	11.10	14.28	13.40		12.92

0.01444	Avg S	10.78	12.95	12.47	0.00834	L.S.D 0.05

**Table 6:** Effect of nano NPK addition and potassium silicate spraying on average seed weight (g) for the 2024 season.

V cultivars	NPK nano (g·L^−1^)	Potassium silicate (mL·L^−1^)			V × N	Avg V
		(0)S1	(2)S2	(4)S3		
Khastawi V1	(0)N1	0.72	0.74	0.67	0.71	0.72
	(2)N2	0.68	0.71	0.71	0.70	
	(4)N3	0.69	0.73	0.78	0.73	

V2 Khadhrawi	(0)N1	0.78	0.70	0.70	0.73	0.71
	(2)N2	0.57	0.77	0.70	0.68	
	(4)N3	0.70	0.70	0.79	0.73	

L.S.D 0.05		0.01499			0.00865	0.00500

V × S	V1	0.70	0.73	0.72	L.S.D 0.05	
	V2	0.68	0.72	0.73	0.00865	

N × S	(0)N1	0.75	0.72	0.68	Avg N	0.72
	(2)N2	0.63	0.74	0.71		0.69
	(4)N3	0.70	0.72	0.79		0.73

0.01060	Avg S	0.69	0.73	0.73	0.00612	L.S.D 0.05

**Table 7:** Effect of nano NPK addition and potassium silicate spraying on average flesh weight (g) for the 2024 season.

V cultivars	NPK nano (g·L^−1^)	Potassium silicate (mL·L^−1^)			V × N	Avg V
		(0)S1	(2)S2	(4)S3		
Khastawi V1	(0)N1	10.04	10.91	10.67	10.54	11.31
	(2)N2	9.96	11.89	13.39	11.74	
	(4)N3	10.12	12.44	12.41	11.66	

V2 Khadhrawi	(0)N1	9.63	10.01	10.34	9.99	11.51
	(2)N2	10.29	13.45	11.82	11.85	
	(4)N3	10.70	14.64	12.75	12.70	

L.S.D 0.05		0.01375			0.00794	0.00458

V × S	V1	10.04	11.75	12.15	L.S.D 0.05	
	V2	10.20	12.69	11.64	0.00794	

N × S	(0)N1	9.83	10.46	10.50	Avg N	10.27
	(2)N2	10.12	12.67	12.60		11.80
	(4)N3	10.41	13.54	12.58		12.18

0.00972	Avg S	10.12	12.22	11.90	0.00561	L.S.D 0.05

**Table 8:** Effect of nano NPK addition and potassium silicate spraying on the average weight of the bunch (kg) for the 2024 season.

V cultivars	NPK nano (g·L^−1^)	Potassium silicate (mL·L^−1^)			V × N	Avg V
		(0)S1	(2)S2	(4)S3		
Khastawi V1	(0)N1	8.43	11.83	12.85	11.04	13.65
	(2)N2	11.97	15.11	16.29	14.46	
	(4)N3	12.42	16.72	17.23	15.46	

V2 Khadhrawi	(0)N1	7.45	10.34	10.85	9.55	12.37
	(2)N2	10.85	14.92	13.46	13.08	
	(4)N3	12.16	16.37	14.90	14.48	

L.S.D 0.05		0.01475			0.00851	0.00492

V × S	V1	10.94	14.55	15.46	L.S.D 0.05	
	V2	10.16	13.88	13.07	0.00851	

N × S	(0)N1	7.94	11.08	11.85	Avg N	10.29
	(2)N2	11.41	15.02	14.88		13.77
	(4)N3	12.29	16.55	16.07		14.97

0.01043	Avg S	10.55	14.21	14.26	0.00602	L.S.D 0.05

**Table 9:** Effect of nano NPK addition and potassium silicate spraying on total yield (kg) for the 2024 season.

V cultivars	NPK nano (g·L^−1^)	Potassium silicate (mL·L^−1^)			V × N	Avg V
		(0)S1	(2)S2	(4)S3		
Khastawi V1	(0)N1	33.71	47.34	51.43	44.16	54.57
	(2)N2	47.54	60.44	65.12	57.70	
	(4)N3	49.69	66.91	68.95	61.85	

V2 Khadhrawi	(0)N1	29.81	41.37	43.43	38.20	49.48
	(2)N2	43.41	59.68	53.89	52.32	
	(4)N3	48.63	65.50	59.59	57.91	

L.S.D 0.05		0.01444			0.00834	0.00481

V × S	V1	43.65	58.23	61.83	L.S.D 0.05	
	V2	40.62	55.52	52.30	0.00834	

N × S	(0)N1	31.76	44.36	47.43	Avg N	41.18
	(2)N2	45.47	60.06	59.50		55.01
	(4)N3	49.16	66.21	64.27		59.88

0.01021	Avg S	42.13	56.87	57.06	0.00589	L.S.D 0.05

**Table 10:** The effect of nano NPK addition and potassium silicate spraying on the percentage of maturity for the 2024 season.

V cultivars	NPK nano (g·L^−1^)	Potassium silicate (mL·L^−1^)			V × N	Avg V
		(0)S1	(2)S2	(4)S3		
Khastawi V1	(0)N1	82.33	89.33	95.33	89.00	84.89
	(2)N2	79.00	81.33	86.00	82.11	
	(4)N3	73.33	85.00	92.33	83.56	

V2 Khadhrawi	(0)N1	82.33	91.33	96.33	90.00	83.67
	(2)N2	76.68	81.33	84.67	80.89	
	(4)N3	70.33	83.33	86.67	80.11	

L.S.D 0.05		0.01441			0.00832	0.00480

V × S	V1	78.22	85.22	91.22	L.S.D 0.05	
	V2	76.45	85.33	89.22	0.00832	

N × S	(0)N1	82.33	90.33	95.83	Avg N	89.50
	(2)N2	77.84	81.33	85.33		81.50
	(4)N3	71.83	84.17	89.50		81.83

0.01019	Avg S	77.33	85.28	90.22	0.00588	L.S.D 0.05

**Table 11:** Estimated cost, revenue, and net income per treatment (per tree basis).

Treatment	Estimated cost (IQD/tree)	Revenue (IQD/tree)	Net income (IQD/tree)	Profitability index %
N1S1 (control)	5000	30000	25000	100% (baseline)
N3S2	12000	54000	42000	168%
N3S3	11000	48000	37000	148%

## Data Availability

The primary data and results presented in this study were obtained from field experiments conducted on date palm trees during the 2024 season and are securely stored by the authors in their complete form. Due to the specific nature of the data and its association with the experimental site, the dataset is not publicly available. However, it can be provided to interested researchers upon submission of a justified request to the corresponding author, in order to ensure proper scientific use and to maintain the confidentiality of the experimental location.
